# Motor excitability is reduced prior to voluntary movements in children and adolescents with Tourette syndrome

**DOI:** 10.1111/j.1748-6653.2012.02033.x

**Published:** 2013-03

**Authors:** Stephen R Jackson, Amy Parkinson, Valentina Manfredi, Guy Millon, Chris Hollis, Georgina M Jackson

**Affiliations:** 1 School of Psychology, University of NottinghamUK; 2 Psychology Department, Pavia UniversityItaly; 3 Division of Psychiatry, University of NottinghamUK

## Abstract

Tourette syndrome (TS) is a neuro-developmental disorder characterized by the occurrence of motor and vocal tics: involuntary, repetitive, stereotyped behaviours that occur with a limited duration, often typically many times in a single day. Previous studies suggest that children and adolescents with TS may undergo compensatory, neuroplastic changes in brain structure and function that help them gain control over their tics. In the current study we used single-pulse and dual-site paired-pulse transcranial magnetic stimulation (TMS), in conjunction with a manual choice reaction time task that induces high levels of inter-manual conflict, to investigate this conjecture in a group of children and adolescents with TS, but without co-morbid Attention Deficit Hyperactivity Disorder (ADHD). We found that performance on the behavioural response-conflict task did not differ between the adolescents with TS and a group of age-matched typically developing individuals. By contrast, our study demonstrated that cortical excitability, as measured by TMS-induced motor-evoked potentials (MEPs), was significantly reduced in the TS group in the period immediately preceding a finger movement. This effect is interpreted as consistent with previous suggestions that the cortical hyper-excitability that may give rise to tics in TS is actively suppressed by cognitive control mechanisms. Finally, we found no reliable evidence for altered patterns of functional inter-hemispheric connectivity in TS. These results provide evidence for compensatory brain reorganization that may underlie the increased self-regulation mechanisms that have been hypothesized to bring about the control of tics during adolescence.

## Background

Tourette syndrome (TS) is a developmental neuropsychiatric disorder that lies at the extreme of the tic disorder spectrum and is characterized by the presence of chronic vocal and motor tics (Leckman, 2002). Tics are involuntary, repetitive, stereotyped behaviours that occur with a limited duration. Motor tics can be simple or complex in appearance, ranging from repetitive movements to coordinated action sequences. Verbal tics can consist of repeating words or utterances (palilalia), producing inappropriate or obscene utterances (coprolalia), or the repetition of another's words (echolalia). Tics occur in bouts, typically many times in a single day, and are the most common form of movement disorder in children (with a prevalence that ranges between 1% and 29% depending upon the precise characteristics of the study population, the diagnostic criteria used, and the study design and methods employed). The etiology of tics is currently poorly understood and most likely involves a complex interaction between genetic and environmental factors that exert an influence over brain development.

The neurological basis of TS is unclear at this time, but it is generally accepted that the basal ganglia, including neural circuits that link the striatum to the frontal lobes, are dysfunctional (Albin & Mink, 2006). Consistent with this proposal, brain imaging and post-mortem studies suggest that there are marked differences in brain anatomy and function in individuals with TS compared to age-matched controls; however, the results of such studies have often been contradictory (for reviews see Gerard & Peterson, 2003; Plessen, Bansal, & Peterson, 2009).

TS typically follows a developmental timecourse (Leckman, 2002) that is associated with increasing control over tics, and appears to be accompanied by compensatory, neuroplastic, alterations in brain structure and function in many individuals with TS (Jackson, Mueller, Hambleton, & Hollis, 2007; Jackson *et al.*, 2011; Mueller, Jackson, Dhalla, Datsopoulos, & Hollis, 2006; Plessen *et al.*, 2004, 2009), but not all. Thus, TS usually first presents during early childhood 3–5 years, and the severity of tics follow a remitting pattern with increasing age. Tic severity is maximal between 11 and 14 years, but tics typically decrease by early adulthood. Importantly, approximately 70–80% of TS sufferers who present with marked tic severity at around 12 years of age have either mild tics or are free of tics by 18 years of age (Leckman, Bloch, Scahill, & King, 2006). Importantly, the majority of individuals with TS appear to develop a means of controlling and effectively suppressing their tics by early adulthood, but a substantial minority continues to have severe tics throughout their adult life. Such control may come about as a result of altered short-range patterns of cortical connectivity (e.g., cortical excitability within a brain area might be altered through changes in the operation of neural circuits, such as inhibitory interneurons, that could function to down-regulate cortical excitability locally either general or in a task-specific manner). Alternatively, enhanced control over motor outputs might arise as a result of altered long-range patterns of cortical connectivity. Thus, it has been suggested that individuals with TS gain control over their tics through the development of compensatory self-regulation mechanisms: most likely implemented through changes in neural pathways linking frontal cortex with primary and secondary motor regions (e.g., Jackson *et al.*, 2011; Makki, Govindan, Wilson, Behen, & Chugani, 2009; Neuner *et al.*, 2010; Plessen *et al.*, 2004). It is possible that altered patterns of control over motor outputs in TS can arise as a result of changed patterns in both long- and short-range cortical connectivity.

One hypothesis proposed by many investigators is that in TS an impairment in the normal operation of basal ganglia-thalamic-cortical circuits gives rise to hyper-excitability of cortical motor areas, which may be brought about by dysfunctional intra-cortical inhibitory mechanisms (e.g., Baumer *et al.*, 2010; Gilbert *et al.*, 2004; Gilbert, Sallee, Zhang, Lipps, & Wassermann, 2005; Moll *et al.*, 1999, 2001; Orth, Munchau, & Rothwell, 2008; Ziemann, Paulus, & Rothenberger, 1997). This hypothesis is supported by recent studies that have made use of transcranial magnetic stimulation (TMS) techniques, particularly paired-pulse TMS (ppTMS), to investigate local alterations in motor cortex excitability in individuals with TS. These studies show considerable consistency, and demonstrate the following. First, resting motor threshold (RMT) and active motor threshold (AMT) do not typically differ between individuals with TS and controls (Heise *et al.*, 2010; Moll *et al.*, 1999, 2001; Orth *et al.*, 2005, 2008; Ziemann *et al.*, 1997; but see Orth & Rothwell, 2009). Second, the duration of the cortical silent period (CSP) induced by TMS to motor cortex, and the magnitude of the short-interval intra-cortical *inhibition* (SICI) that is observed, are both significantly reduced in individuals with TS relative to matched controls (e.g., Gilbert *et al.*, 2004; Moll *et al.*, 1999, 2001; Orth *et al.*, 2008; Ziemann *et al.*, 1997). Importantly, the reduction in SICI observed in individuals with TS has been shown to correlate with ADHD scores in individuals with TS (e.g., Gilbert *et al.*, 2004, 2005) and with clinical measures of tic severity (e.g., Gilbert *et al.*, 2004; Orth *et al.*, 2008). Specifically, reduced SICI magnitude is linearly associated with increased tic severity. Third, intra-cortical *facilitation* appears to be unaffected in TS (e.g., Moll *et al.*, 2001; Ziemann *et al.*, 1997) and is uncorrelated with clinical symptoms (Gilbert *et al.*, 2004).

The studies referred to above provide good evidence for a reduction in intra-cortical inhibition and hyper-excitability within motor cortex in TS that is correlated with clinical measurements of tic severity. However, these effects may be modulated by compensatory, neuroplastic, changes in the structure and function of longer range cortical circuits, including inter-hemispheric connectivity and connections between frontal and motor areas. Examples may include alterations in the white-matter microstructure of neural pathways linked to motor areas (Jackson *et al.*, 2011; Neuner *et al.*, 2010; Plessen *et al.*, 2004, 2006); and recruitment of additional cortical regions (e.g., prefrontal cortex) to aid in the control of motor outputs (Jackson *et al.*, 2011), perhaps by ‘dampening’ the hyper-excitability of motor areas. Evidence in support of this idea comes from a recent fMRI study in which the Blood Oxygenation Level Dependent (BOLD) signal recorded in the motor cortex of individuals with TS was significantly *reduced* relative to controls (who were performing at the same level on a behavioural task), whereas the BOLD signal recording in the right prefrontal cortex of the TS group was significantly *increased* relative to controls, and, in the TS group only, was significantly correlated with performance on the behavioural task (Jackson *et al.*, 2011).

Two recent TMS studies are also of particular note in this context: first, Heise *et al.* (2010) used ppTMS to investigate the modulation of motor cortex excitability during the preparation period that preceded a voluntary movement. These showed that while RMT and AMT did not differ between controls and individuals with TS, the TS patients showed less of an increase in motor-evoked potential (MEP) amplitude prior to movement onset than controls. That is, motor excitability immediately prior to a voluntary movement was in fact significantly reduced in the TS group relative to controls. This finding is consistent with the notion that in many individuals with TS, motor cortex excitability may be held in check by increased cognitive control mechanisms (Jackson *et al.*, 2007; Jackson *et al.*, 2011; Mueller *et al.*, 2006). Second Baumer *et al.* (2010) combined Diffusion Tensor Imaging (DTI) and dual-site ppTMS (ds-ppTMS) techniques to investigate inter-hemisheric connectivity between the primary motor cortices of adults with pure TS. They found that in TS patients the magnitude of left-to-right inter-hemispheric cortical inhibition was weaker than that observed in controls. Furthermore, they noted a significant *negative* linear relationship between the white-matter microstructure and left-to-right Inter-hemisperic-inhibition (IHI) in the control group only, suggesting that individuals with TS have altered inter-hemispheric connectivity between primary motor areas.

In the current study we independently investigate the issue reported upon by Heise *et al.* (2010), by examining, in children with ‘pure’ TS, whether TMS delivered immediately prior to a movement results in significantly reduced cortical excitability relative to control subjects. Specifically, we delivered TMS during the pre-movement phase of a version of the manual choice reaction time task used by Boorman, O'Shea, Sebastian, Rushworth, and Johansen-Berg (2007) that involves responding, using either the right or left hand, according to the colour/shape attributes of a visual cue. This task induces high levels of inter-manual conflict (Boorman *et al.*, 2007) that is likely resolved in typically developing individuals through inter-hemispheric communication via the corpus callosum. However, this may not necessarily be the case in individuals with TS (see Jackson *et al.*, 2011).

One issue raised by our use of the above task is the assumed relationship between such tasks and the occurrence of tics in TS. This is obviously a complex issue that requires further study; however, we take the view that the motor structures (e.g., primary motor cortex, SMA, etc.) that are involved in the planning and execution of voluntary actions are also strongly implicated in the generation of tics in TS (Bohlhalter *et al.*, 2006; Mantovani *et al.*, 2006) and that physiological or brain-imaging measures of functional activity in these regions has previously been shown to be a significant predictor of clinical measures of tic severity (e.g., Gilbert *et al.*, 2004; Jackson *et al.*, 2011; Orth *et al.*, 2008). Furthermore, while it may be of interest to compare the performance of the TS group on the behavioural choice reaction time task, relative to typically developing controls, it is important to note that this is not the focus of this paper and to recognize that the behavioural task is included here solely to induce, in all individuals, a state of motor preparedness when TMS is delivered to the motor cortex.

We also make use of ds-ppTMS to investigate directly whether there are differences in long-range cortical connectivity in TS. This technique involves delivering a single, above-threshold, TMS pulse, of a fixed magnitude, to one brain area (referred to as the ‘test’ stimulus), and investigating how the effects of the test stimulus are modified by the delivery of a sub-threshold TMS pulse (referred to as the ‘conditioning’ stimulus) which is delivered several milliseconds before the test stimulus to a different but interconnected brain area. Using this technique, it is possible to investigate both inhibitory and facilitatory modulation effects of one brain area on another. Specifically, as the manual choice reaction time task used by Boorman *et al.* (2007) has been shown to induce high levels of inter-manual conflict (Boorman *et al.*, 2007) that is likely resolved in typically developing individuals through inter-hemispheric communication via the corpus callosum, we investigated directly whether there are altered patterns of *inter-hemispheric* modulation from the primary motor cortex in one hemisphere on the contralateral primary motor cortex, in a group on adolescents with TS compared to a group of matched control individuals.

In the current study, our clinical sample was restricted to a group of children and adolescents with TS who presented without co-morbid ADHD. In this respect our sample differs from that reported in the study by Baumer *et al.* (2010). Excluding individuals with co-morbid disorders such as ADHD is sensible when trying to investigate the behavioural correlates of TS, however it should be noted that such samples may not necessarily reflect individuals who present at a TS clinic, who may more typically present with co-morbidities.

## Methods

### Subjects

Ten right-handed adolescents (9 males; mean age 14 years, 7 months), with a current diagnosis of TS were recruited for this study. A control group was recruited that compromised 12 typically developing, age-matched, individuals (11 males; average age 14 years, 2 months). All subjects were right-handed. Patients were recruited through the TS clinic at the Queens Medical Centre, Nottingham. Participants who had a clinical diagnosis of ADHD or OCD were excluded from the sample. Current tic severity for the TS patients was assessed on the day of testing using the Yale Global Tics Severity Scale (Leckman *et al.*, 1989). The IQ of all participants was obtained using the Wechsler Abbreviated Scale of Intelligence (WASI) vocabulary and matrix reasoning subscales. Preliminary analyses confirmed that the IQ of the TS and control groups did not differ. Clinical data from the patient group are summarized in Table 1. Approval for all procedures reported here was obtained from the Nottingham Healthcare Trust, and informed written consent was obtained from all individuals prior to participation.

**Table 1:** Clinical details of TS group

ID	Years	Months	M/F	IQ	Global YGTSS	Motor YGTSS	Medication
TS01	17	1	M	76	30	11	Clonidine 50 μg
TS02	12	5	M	103	14	0	None
TS03	14	7	M	120	28	10	None
TS04	20	10	M	112	55	15	Risperidone 1.5 mg
TS05	13	7	M	135	25	11	Clonidine 50 μg
TS06	14	7	M	111	21	14	None
TS07	15	8	F	95	7	7	Clonidine 75 μg
TS08	13	7	M	101	18	10	None
TS09	10	10	M	118	29	12	None
TS10	16	4	M	111	21	11	Clonidine 75 μg

*Note*. YGTSS, Yale Global Tic Severity Scale.

### Apparatus

The behavioural task used in this study was modelled on that reported by Boorman *et al.* (2007). Stimuli were displayed on a 17-in. computer monitor with a spatial resolution of 640 × 480 pixels at a frame rate of 60 Hz, at a viewing distance of 46 cm. Stimuli were generated using the MATLAB Cogent Graphics toolbox developed by John Romaya at the Wellcome Department of Imaging Neuroscience. MATLAB code was also used throughout to trigger the delivery of TMS, record electromyography (EMG) data, and record the subject's responses and reaction time. Participants responded to on-screen stimuli with a custom-built button box containing two micro-switches. A chinrest was used to minimize unwanted head movements.

The functional ‘hot spot’ corresponding to the hand region of the right and left primary motor cortex was initially found for each subject through a trial-and-error process of hunting for a robust and consistent TMS-induced muscle twitch in the first dorsal interosseous (FDI) muscle of the contralateral hand.

Two separate Magstim, Rapid 200, TMS monophasic stimulator units (Magstim Ltd., UK), each equipped with multiple 50-mm figure of eight coils, were used to deliver TMS. Manfrotto clamps were used to position and secure the TMS coils in place on the appropriate scalp location. EMG data were collected using a G.tec g.USB biosignal amplifier that had a sampling frequency of 256 Hz. Active and passive Ag/AgCl surface electrodes with a diameter of 7 mm were attached to the FDI muscle in the right hand, while a ground electrode was attached to the styloid process of the right wrist.

### TMS

The procedures for localizing the hand motor areas and for obtaining RMT are based on those reported by Boorman *et al.* (2007). First, to find the hand area of the left motor cortex we measured the distance between the nasion and inion, and marked the midpoint between these two points as the vertex (position Cz according to the 10–20 EEG coordinate system). We then measured 3 cm laterally from the vertex towards the left ear and we initially positioned the coil in this position. We moved the coil in 0.5-cm steps around this area in order to find the ‘hot spot’, which elicited the largest MEP responses in the contralateral FDI muscle of the hand when stimulated, and we marked this point as the hand area of the left motor cortex. The same procedure was used to find the hand area of the right motor cortex, but in this case we measured 3 cm lateral on the right from the vertex. As noted above, each hand area ‘hot spot’ was checked against anatomical landmarks.

Once the hand motor area in each hemisphere had been appropriately localized, we obtained RMT for each hemisphere. Again, we followed the procedures described in Boorman *et al.* (2007) to obtain RMT. First, to find the RMT of the left motor cortex, we delivered single TMS pulses to the hot spot of left the motor cortex and measured MEPs in the FDI of the contralateral hand. Pulses were initially delivered with a low TMS intensity (∼40% stimulator output) and intensity was increased until a muscle twitch was observed in the contralateral FDI that corresponded to an MEP of ∼1 mV. We then chose the minimum TMS intensity required to obtain a discernable muscle twitch (∼1 mV) in the contralateral FDI when the subject's hand was relaxed, in at least 50% of 10 consecutive stimulations. The same procedure but then used to find the RMT of the right motor cortex. Once localization of each hand motor area was complete, and RMT had been obtained for each hemisphere, a TMS coil was placed over the hand area of each motor cortex.

**Figure 1:**
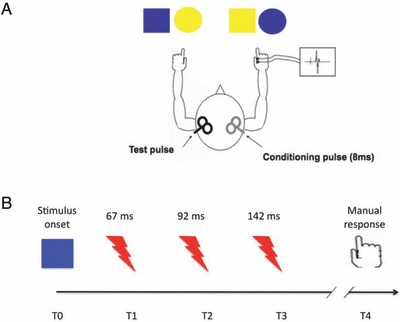
(A) Schematic illustration of an example mapping of the visual stimuli to each hand in the behavioural choice reaction time task, and the location and orientation of the TMS coils in the dual-site paired-pulse TMS condition. (B) Schematic representation illustrating the timecourse of TMS delivery within the period between the onset of the visual stimulus and the participant executing a response. The TMS test pulse could be delivered with an ISI of 67, 92, or 142 ms.

Two TMS protocols were used in the current study: single-pulse TMS (spTMS) and a ds-ppTMS protocol. In the spTMS protocol, the coil placed over left motor cortex delivered a single TMS pulse 67, 92, or 142 ms after the onset of the visual stimulus, but prior to movement onset. These times were chosen based upon the results reported by Boorman *et al.* (2007). The intensity of this single pulse (sp) was set to 120% of the RMT for that individual. In the ds-ppTMS protocol the coil placed over the hand area of the left motor cortex delivered an identical pulse (hereafter referred to as the test pulse), at identical times, as described above for the spTMS trials (Figure 1A). However, the coil placed over the hand area of the right motor cortex delivered a sub-threshold single TMS pulse (hereafter referred to as the conditioning pulse) that preceded the onset of the left hemisphere test pulse by an inter-stimulus interval (ISI) of 8 ms. The intensity of this conditioning pulse was set at 90% of RMT.

For those unfamiliar with the technique, the rational for using the ds-ppTMS paradigm to investigate the efficacy of inter-hemispheric communication is as follows. A single ‘test’ pulse of TMS delivered at or above threshold to the hand area of motor cortex will be sufficient to produce a measurable change in the MEP measured from muscles in the hand. The efficacy of inter-hemispheric transfer can then be studied by investigating by how much the magnitude of the MEP that follows this test pulse is altered by delivering another ‘conditioning’ TMS pulse to the contralateral hand motor area shortly before the delivery of the test pulse.

### Procedure

Participants were seated in front of a computer screen aligned with the subject's sagittal midline with the head immobilized by a chin rest. Visual stimuli consisted of yellow circles, blue squares, blue circles, and yellow squares that had a diameter of 2 cm (Figure 1A). Two of these stimuli (e.g., yellow circle and blue square) were mapped to one-hand response and the remaining two stimuli were mapped to the other hand. This mapping ensured that neither stimulus colour or stimulus shape were alone sufficient to determine the correct response. The order of mapping was randomized across individual participants. Stimuli appeared in a random order in the middle of the screen in a white background and the subject was asked to respond as quickly as possible. A total of 90 stimuli were presented in total

Each trial started with a fixation cross that was presented in the centre of the computer screen. Recording of 5 s of EMG commenced with the onset of the fixation cross. After a variable period (mean = 1,500 ms; *SD*= 39 ms), a single-visual stimulus was drawn randomly from the set of possible targets, and presented in the centre of the computer screen. This stimulus remained visible until the subject made a manual keypress response or until the trial timed out after 2 seconds (Figure 1B). Prior to the start of the experiment each participant completed 40 practice trials. In addition, the first 10 trials of the experiment were treated as additional practice trials and were discarded from the analyses.

As noted above, the stimulus onset asynchrony between the fixation cross and the main TMS pulse was variable. In a third of the trials it was 67 ms, in another third of the trials it was 92 ms, and in the remaining third it was 142 ms. The timing between onset of visual stimuli and the first TMS pulse was kept sufficiently short so that the participants could not respond before the TMS had been delivered (Figure 1B).

## Results

### Preliminary analyses

Preliminary analyses were initially carried out to determine whether the groups differed in their performance of the response-conflict task. In these analyses, responses were analysed across *both* hands, and were not restricted solely to responses made with the hand contra-lateral to the test TMS pulse (as is the case for later analyses reported below). As the number of errors made on this task was small (>3%) only correct response time (RT) trials are reported and error trials were not analysed further.

Median RTs were calculated separately for each condition and for each individual. The means RT for each condition, and for each group, are presented in Table 2. These data suggest comparable performance across the different conditions (i.e., No TMS, spTMS, ds-ppTMS) and between groups. This was tested using a mixed ANOVA with group as the between-subject factor and condition the within-subject factor. The ANOVA revealed no significant main (*F*[1,20] < 1, *p* > .1) or interaction (*F*[2,40] < 1, *p* > .1) effects. Importantly, this result confirms that the individuals with TS were performing the response-conflict task at the same level as the controls, and confirms many previous studies reporting that individuals with TS are not impaired on cognitive control tasks relative to age-matched typically developing individuals (e.g., Mostofsky, Lasker, Singer, Denckla, & Zee, 2001; Ozonoff, Strayer, McMahon, & Filloux, 1994; Ozonoff, Strayer, McMahon, & Filloux, 1998).

**Table 2:** Mean of median response times (milliseconds) for both left and right hand responses for each TMS condition

Group		Condition		
	
		No TMS	Single-pulse TMS	Double-pulse TMS
Control group	Mean	675	653	669
	*SD*	97	110	113
Tourette group	Mean	698	700	684
	*SD*	118	118	92

**Figure 2:**
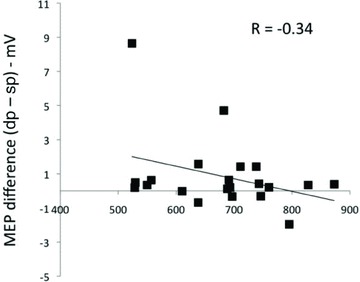
Scatter plot together with regression line illustrating, for all subjects, the linear relationship between median RT of right-hand behavioural responses and the difference in MEP amplitude between double-pulse and single-pulse TMS trials. The figure demonstrates a modest negative correlation (*R*=−.34) indicating that larger MEP difference scores (increased modulation by the conditioning TMS pulse) are associated with faster RT scores.

To examine whether participants’ RT responses were predicted by individual differences in the physiological efficiency of inter-hemispheric connections between the hand motor areas, we examined the linear relationship between RT in the No TMS condition and the size of the modulation in the observed MEP obtained from the right hand FDI brought about by an ipsilateral conditioning TMS pulse (i.e., dual-pulse TMS MEP–spTMS MEP). These data are presented in Figure 2 that shows a modest negative correlation (*R*=−.34, *p* > .1), indicating that shorter RTs were associated with increased ds-ppTMS modulation of MEP. While there is an indication that this association may be stronger in controls (*R*=−.43) compared to the TS group (*R*=−.23), this difference was not statistically significant (*Z*=−0.45).

### Analyses of data from contralateral hand

#### RT data

To explore the data further, we examined only behavioural responses made using the right hand and MEP responses obtained from the FDI of the right hand. Note that the right hand was contralateral to the test TMS pulse and ipsilateral to the conditioning TMS pulse on dpTMS trials and contralateral to the TMS pulse on spTMS trials.

**Figure 3:**
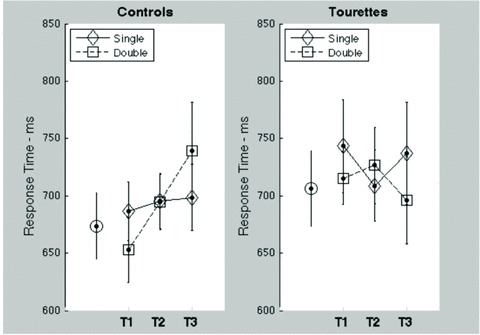
Mean response times for right-hand responses for Tourette syndrome participants and controls. Data are presented separately for No TMS trials (open circle symbol), single-pulse, and double-pulse trials, and for each ISI (T1–T3). Error bars are standard error of the mean.

To explore the effect of delivering TMS during the pre-movement period on RT, we examined mean RT for each group in the No TMS condition compared to when a sp of TMS was delivered at T1 (67 ms), T2 (92 ms), or T3 (142 ms) after stimulus onset. These data are presented in Figure 3 and differences between means were examined using a mixed ANOVA with group as the between-subject factor and condition (No TMS, T1, T2, T3) the within-subject factor. The ANOVA revealed no significant main (*F*[1,20] < 1, *p* > .1) or group × condition interaction effects (*F*[3,60] < 1, *p* > .1). This result indicates that spTMS had no effect on the latency of manual responses.

#### Analyses of MEP data

Median MEPs from the FDI muscle of the right hand were calculated for each participant for each ISI (67, 92, 142 ms) and each condition (sp vs. dp). Preliminary analyses revealed that baseline MEP, in the absence of stimulation, did not differ between the TS group and controls (*p*= .67).

### Pre-movement reduction of MEP in TS group

In their recent paper, Heise *et al.* (2010) demonstrated that although RMT and AMT did not differ between adults with TS and controls, in the period preceding movement onset the TS group exhibited a significantly reduction in the amplitude of TMS-induced cortical excitability relative to controls. We investigated this effect directly in the current study.

**Figure 4:**
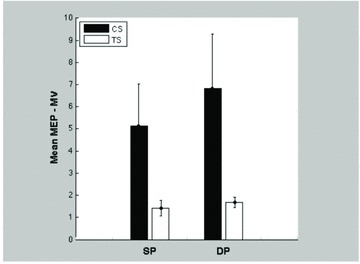
Mean MEP amplitude for each type of TMS trial (single pulse [sp] vs. double pulse [dp]) and for each group (Tourettes vs. controls). Error bars are standard error of the mean. The difference in MEP between groups is statistically significant for both types of TMS stimulation (*p* < .05).

First, having verified, using an ANOVA, that there was no significant difference between trials with different ISI values (*F*[2,40]= 1.3, *p*= .28), we collapsed the data across the different ISIs to produce, for each subject, a mean MEP value for each TMS condition (i.e., sp vs. dp trials). These data are presented in Figure 4.

We investigated the simple effects of *group* for each TMS condition in a set of planned contrasts based upon independent *t*-tests. These analyses confirmed that group had a statistically significant effect in both the sp (means: control group = 5.14 mV, Tourette group = 1.41 mV; *t*(20) = 1.78, *p* < .05; effect size = 0.56) and dp (means: control group = 6.82 mV, Tourette group = 1.68 mV; *t*(20) = 1.90, *p* < .05; effect size = 0.6) trials. These data provide an important replication of the effect reported by Heise *et al.* (2010) in adults with TS, insofar as they demonstrate that young adolescents with TS also exhibit a reduction in cortical excitability, relative to controls, during the period leading up to the execution of a movement.

### Inter-hemispheric modulation of MEP amplitude

Mean MEPs for each group (controls vs. Tourettes), TMS condition (spTMS vs. dpTMS), and ISI (67, 92, and 142 ms) are presented in Table 3. As noted above, preliminary analyses (ANOVA) had confirmed that there were no significant differences across the different ISI values (*F*[2,40]= 1.3, *p*= .28). Data were therefore collapsed across the different ISIs.

**Table 3:** Mean of median MEP (mV) obtained from the FDI muscle of the right hand in the period preceding movement onset for each TMS condition and ISI

Group		Condition					
	
		Single-pulse TMS			Double-pulse TMS		
		
		ISI 67 ms	ISI 92 ms	ISI 142 ms	ISI 67 ms	ISI 92 ms	ISI 142 ms
Control group	Mean	6.60	3.76	5.07	5.71	7.43	7.31
	*SD*	9.22	4.14	6.54	5.42	9.31	11.13
Tourette group	Mean	1.89	1.22	1.14	1.69	1.55	1.80
	*SD*	2.09	.80	.79	.72	.91	.81

The effect of TMS condition (spTMS vs. dpTMS) was assessed for each group separately in planned contrasts using paired *t*-tests. The results of these analyses revealed that whereas MEPs were significantly larger on dp trials compared to sp trials for the control group (means: sp = 5.14 mV, dp = 6.82 mV; *t*(11) =−1.843, *p* < .05; effect size = 0.22), they did not differ in the Tourette group (means: sp = 1.41 mV, dp = 1.68 mV; *t*(9) < 0.1, *p* > .1).

In previous studies making use of the ds-ppTMS paradigm, the modulation effect that a conditioning pulse delivered to one hemisphere has on a test pulse delivered to the opposite hemisphere, has been assessed by computing, for each participant, a difference or ratio measure of the MEP obtained on sp and dp trials. We computed three such measures in the current study: the difference between MEPs for dp and sp trials (i.e., dp − sp); the ratio of MEPs for dp and sp trials (i.e., dp/sp); and, difference between MEPs for dp and sp trials expressed as a proportion of spMEP (i.e. (dp − sp)/sp). Each of these measures suggested that the magnitude of modulation caused by dpTMS was greater in the control group than in the TS group. However, the statistical analyses failed to confirm this as analysis of each measure using independent group *t*-tests revealed that there were no significant effects of group on any measure (maximum *t*(20) = 1.37, *p*= .09).

## Discussion

We used sp and ds-ppTMS to investigate altered patterns of cortical excitability in a sample of adolescents and young adults with TS who were engaged in executing a manual task that involved high levels of inter-manual conflict. Specifically, we examined two issues: first, do individuals with TS exhibit decreased motor cortical excitability in the period immediately preceding the onset of a movement? Second, do individuals with TS show altered patterns of inter-hemispheric functional connectivity, as indexed by changes in MEP amplitude in response to a conditioning pulse of ipsilateral TMS.

The main findings from this study are as follows: first, performance on the behavioural response-conflict task was equivalent for the adolescents with TS and the age-matched control group; second, while pre-stimulation FDI MEPs for the TS and control groups did not differ from one another, the TS group showed reduced MEP amplitudes during the period immediately preceding a movement, when individuals would presumably be engaged in response selection and movement preparation; third, comparison of a range of dependent measures revealed little evidence for altered *inter-hemispheric* function in adolescents with TS. These findings are discussed below.

It is a widely held belief that individuals with TS are impaired on tasks that index executive function or cognitive control. This belief appears to be predicated largely on the premise that, because those with TS cannot easily suppress their tics, they must have difficulties with the cognitive control of their behaviour, and in particular with inhibiting task-irrelevant responses or stimuli in laboratory tasks of executive function. However, the existing empirical evidence does not readily support this view. Thus, while there have been a number of studies that have reported impaired executive function in TS (e.g., Channon, Flynn, & Robertson, 1992; Harris *et al.*, 1995; Sherman, Shepard, Joschko, & Freeman, 1998; Swerdlow, Magulac, Filion, & Zinner, 1996), there have been as many studies that have reported no differences between groups (e.g., Li, Chang, Hsu, Wang, & Ko, 2006; Mostofsky *et al.*, 2001; Ozonoff *et al.*, 1994, 1998; Yuen, Bradshaw, Sheppard, Lee, & Georgiou-Karistianis, 2005), and also studies that have demonstrated enhanced cognitive control in individuals with TS (e.g., Jackson *et al.*, 2007, 2011; Mueller *et al.*, 2006). As noted above, it is likely that the inconsistency in behavioural findings arises as a consequence of a number of factors, which may include: the use of a wide range of behavioural tasks to assess executive function, the execution of which draw on different cognitive processes, and of which only some may be compromised in TS; the inclusion in many studies of adults with TS or mixed samples of children and adults; and, the failure in many studies to exclude individuals presenting with co-morbid disorders such as ADHD, which are themselves highly associated with executive dysfunction.

The current study made use of a cognitively demanding manual keypress task that induces high levels of inter-hemispheric conflict (Boorman *et al.*, 2007) and demonstrated that the adolescents with TS performed the task to the same level as that observed for a group of age-matched typically developing controls. As such this confirms that studies that consist of children or adolescents with TS, who present without co-morbid ADHD, rarely demonstrate impairments in cognitive control in TS, and very often instead report enhancements in the cognitive control of behaviour (e.g., Jackson *et al.*, 2007, 2011; Mueller *et al.*, 2006).

### Reduced cortical excitability in TS preceding a movement

We demonstrated that whereas pre-stimulation FDI MEPs did not differ between the TS and control groups, the TS group exhibited significantly reduced MEP amplitudes in response to a TMS pulse delivered during the period immediately preceding the execution of a finger movement. Furthermore, it should be kept in mind that this reduction in pre-movement MEP relative to controls was observed in the absence of any reduction in behavioural performance.

This finding is important for a number of reasons. First, it replicates in a sample of adolescents with TS, the effect recently reported by Heise *et al.* (2010) in a sample of adults with TS, in which adults with TS were clearly shown to exhibit reduced MEPs preceding a movement. Second, it provides supporting evidence for the suggestion that control over motor tics may come about through the active suppression of hyper-excitable motor cortex (Jackson *et al.*, 2007; Jackson *et al.*, 2011; Mueller *et al.*, 2006). Specifically, to avoid the expression of unwanted movements, motor cortex may be actively suppressed by way of cognitive control mechanisms (likely involving prefrontal cortex) during periods of movement selection or planning. Consistent with this view, we have recently shown that, during the execution of a manual response-conflict task that involved high levels of inter-manual conflict, the fMRI BOLD response in the hand area of motor cortex was significantly *reduced* in a group of adolescents with TS relative to age-matched controls, despite performance on the behavioural task being equivalent across the groups (Jackson *et al.*, 2011). The idea that control over tics in TS may be brought about by adaptive changes in which hyper-excitable motor cortex is gradually brought under control through functional or structural brain changes is also consistent with the many reports of changes in white-matter microstructure within the TS brain; particularly reports of thinning of corpus callosum (e.g., Plessen *et al.*, 2004) and reduced FA/increased diffusivity in the corpus callosum (e.g., Jackson *et al.*, 2011; Neuner *et al.*, 2010).

### Alterations in inter-hemispheric functional connectivity in TS

Previous reports of altered patterns of white-matter microstructure in the corpus callosum (e.g., Plessen *et al.*, 2004) suggested that motor cortical hyper-excitability might be controlled through changes in inter-hemispheric functional connectivity between primary motor cortices. Thus, it was something of a surprise to us that in the current study, we could find no reliable evidence for altered functional connectivity in the TS group using the ds-ppTMS technique. Particularly as a recent study reported by Baumer *et al.* (2010) used this technique to demonstrate reduced left-to-right inter-hemispheric cortical inhibition (IHI), relative to controls, in a group of unmedicated adults with TS but without co-morbid disorders.

Several factors may have led to the absence of an effect in the current study. These are discussed in turn. First, it should be acknowledged that the evidence that control of motor cortical excitability in TS might be based upon altered inter-hemispheric functional connectivity is not well developed. Thus, the bulk of the existing TMS studies have used ppTMS to study *intra-cortical* inhibition effects, and have reported reduced levels of intra-cortical inhibition which may be linked to altered functioning of gamma-aminobutyric acid (GABA)-mediated intra-cortical circuitry (e.g., Heise *et al.*, 2010). It may well be that altered patterns of control of hyper-excitable motor cortex occurs primarily via modulation by ipsilateral connections from higher order premotor and prefrontal regions. Second, the findings of the Baumer *et al.* (2010) study are not entirely straightforward to interpret as these authors only found a between-group difference for left-to-right IHIs; that is, when the test pulse was delivered to the right motor cortex and the conditioning pulse to the left there was an effect. In their study, right-to-left IHI in the TS group was comparable to that of the controls. In the current study, as all participants were right-handed, we delivered the test pulse to the cortex controlling the right hand and therefore investigated only right-to-left modulation effects. It remains to be seen if left-to-right IHI is impaired in our sample. Third, the Baumer *et al.* (2010) study investigated adult participants with TS. As outlined above, there is very good evidence that adults with TS are unrepresentative of children and adolescents with TS, and that effects observed in adults are very often not observed in children (see Gerard & Peterson, 2003; Plessen *et al.*, 2009 for recent reviews). It is entirely possible that the effect observed in the above study is confined to individuals whose TS persists into adulthood, or else develops over the course of adolescence and thus is incomplete in our sample. Fourth, in the current study, we followed Boorman *et al.* (2007) in using an 8-ms inter-stimulus interval between the conditioning and test pulse on ds-ppTMS trials. This interval is entirely consistent with previous studies that have reported inter-hemispheric inhibition using ISIs between 6 and 15 ms (e.g., Ferbert *et al.*, 1992), however in the current study, we observed that at the level of individual subjects, the difference in median MEPs between dp and sp trials was not always negative (consistent with inter-hemispheric inhibition), and in fact the overall difference in means at the group level was positive (consistent with an inter-hemispheric facilitation effect). As ppTMS studies have previously reported that *intra-cortical* facilitation is not different in individuals with TS relative to matched controls, it may be useful in future studies to explore ds-ppTMS effects in TS using a range of ISIs that extend to 15 ms or longer and are more likely to yield unambiguous inter-hemisphere inhibition.

In summary, we used a ds-ppTMS protocol, together with a manual RT task that involved high levels of inter-manual conflict, to explore motor cortical excitability in the immediate period preceding a finger movement, and inter-hemispheric functional connectivity between the hand motor areas, in a group of adolescents with TS, but without co-morbid ADHD. The main findings of the study were that performance on the behavioural response-conflict task did not differ in the adolescents with TS, despite the fact that the excitability of their motor cortex, as measured by TMS-induced MEPs in the period preceding execution of a motor response, was significantly reduced compared to typically developing individuals. We found no evidence of differences in functional inter-hemispheric connectivity in the TS group relative to typically developing adolescents.
